# The rise of fungal G-protein coupled receptors in pathogenesis and symbiosis

**DOI:** 10.1371/journal.ppat.1013212

**Published:** 2025-06-12

**Authors:** Max Heinen, Hanna Rovenich, Florian Altegoer

**Affiliations:** 1 Institute of Microbiology, Heinrich-Heine-University, Cluster of Excellence in Plant Sciences (CEPLAS), Düsseldorf, Germany; 2 Institute for Plant Sciences, Cologne University, Cologne, Germany; University of Maryland, Baltimore, UNITED STATES OF AMERICA

G-protein coupled receptors (GPCRs) are key regulators in coordinating environmental sensing with intracellular responses. While their roles in signaling and development are well understood in mammals, fungal GPCRs remain largely unexplored. Key information on receptor classification, ligand activation, and G-protein interactions is still limited. However, recent advances in combining genomics, metagenomics, and synthetic biology with structural biology—especially structure prediction and cryo-electron microscopy (cryo-EM) —are opening new avenues for studying fungal GPCRs. In this review, we highlight recent discoveries on how GPCRs mediate fungal interactions, explore their potential roles in complex microbial communities, and suggest future research directions in this emerging field.

## Introduction

G-protein coupled receptors (GPCRs) are the largest class of cell surface receptors in eukaryotes and have been extensively studied in humans. They are characterized by a seven-transmembrane (7TM) helical fold and translate extracellular signals into intracellular responses. At the intracellular interface, most GPCRs are associated with heterotrimeric G-proteins that dissociate upon ligand binding and interact with two major signaling pathways involving cAMP-protein kinase A (PKA) and mitogen-activated protein kinase (MAPK) cascades. These pathways have been studied for decades and are well-understood in several fungal species [1]. Notably, GPCRs can also be coupled to alternative regulators such as arrestins but our understanding of their role in fungi remains limited [2].

Fungal GPCRs exhibit a greater sequence and domain diversity than their mammalian counterparts [1]. The mammalian GPCR family is subdivided into six classes, while GPCRs of >1300 fungal species have recently been categorized into 17 classes, including only four of the six mammalian GPCR classes (Rhodopsin, Glutamate, Frizzled, and Secretin) present in fungi [3].

The identified GPCR classes are not equally distributed among fungal species. For example, the baker’s yeast *Saccharomyces cerevisiae* harbors only three GPCRs, which are also among the best-studied examples of fungal GPCRs, while the genomes of some filamentous fungi contain up to 100 GPCRs of various classes [3]. Despite the growing catalog of fungal GPCRs, the vast majority remain poorly characterized. For most, key information on their activating ligands, mechanisms of activation, and associated signaling components is still lacking. This functional gap is becoming increasingly pronounced as genome sequencing efforts continue to identify novel GPCR candidates. In addition, the large number and diversity of these receptors suggest that they may mediate a wide range of physiological and ecological responses, many of which are yet to be uncovered.

In this review, we provide a comprehensive overview of the current state of knowledge on fungal GPCRs, with a particular focus on a set of well-studied examples. We examine how these receptors regulate key processes underlying fungal adaptation and survival across different ecological contexts—including in microbial communities such as lichens. We also discuss how GPCR diversification facilitates the recognition of a broad range of ligands and the engagement of novel signaling mechanisms. Finally, we highlight how advances in genomic analyses, structural modeling, and synthetic biology are expanding the toolkit for GPCR discovery and characterization, paving the way for exploiting GPCRs as potential targets in fungal biotechnology and antifungal strategies.

## GPCRs regulate central aspects of fungal adaptation and survival

**Fig 1:**
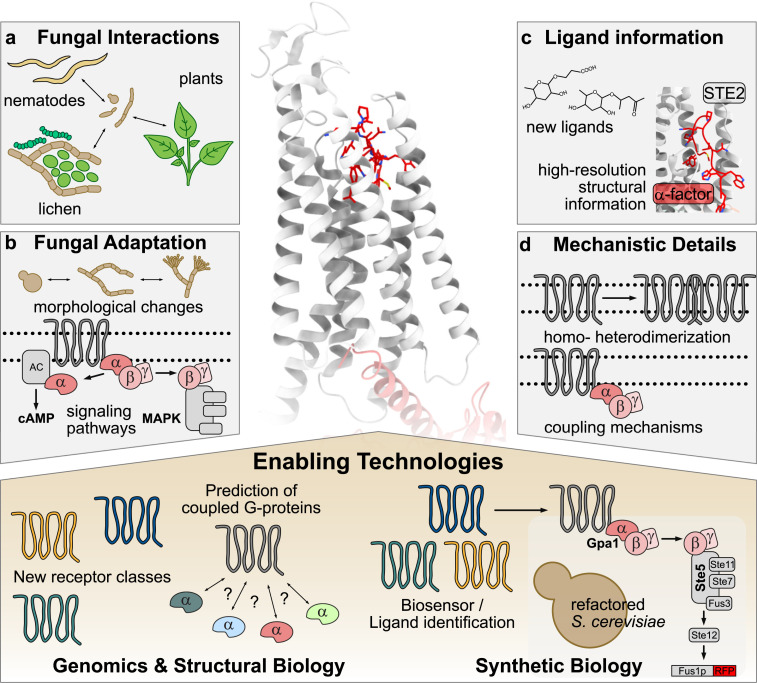
Advances in fungal G-protein coupled receptor (GPCR) research and key findings. (Center) Representative fungal GPCR: First structural complex of *Saccharomyces cerevisiae* Ste2 with the α-pheromone peptide ligand and Gpa1 (PDB-code:7AD3) elucidated by cryo-electron microscopy. **(a)** GPCRs mediate a broad spectrum of fungal interactions with various organisms including nematodes, plants, and within microbial communities such as lichens. **(b)** Conserved GPCR-triggered cAMP and mitogen-activated protein kinase (MAPK) signaling pathways regulate morphological changes and other cellular processes to facilitate fungal adaptation to different environments in diverse ecological contexts (see a). **(c)** Structural insights into fungal GPCRs reveal ligand identities and their recognition mechanisms. **(d)** Some fungal GPCRs exhibit homo- and heterodimerization and can couple to G-proteins and other signaling components in different ways. (Bottom) Genomics, structural biology, and synthetic biology facilitate the identification of new receptor classes, prediction of coupled G-proteins, and in-depth characterization of ligands.

GPCRs facilitate the communication between fungi and their environment and are particularly important for diverse adaptation processes promoting fungal survival and propagation during interactions with hosts, prey, and symbiotic partners (Fig 1a) [1, 4–6]. Mating is perhaps the best-studied GPCR-mediated process in fungi. In *S. cerevisiae*, the mating GPCRs Sterile2 (Ste2) and Sterile3 (Ste3) detect peptide pheromones from compatible partners, triggering a MAPK cascade that induces cell cycle arrest, morphological changes such as polarized growth (shmoo formation), and transcriptional changes necessary for cell fusion and mating-type switching [7] (Fig 1b). In some pathogenic fungi, sexual reproduction is tightly linked to host colonization and triggers cellular programs required for virulence, while it is dispensable in other pathogens [8]. This illustrates that, while the core molecular signaling components are largely conserved across species, fungi have evolved remarkable plasticity in their mating behaviors and downstream adaptive strategies.

Besides mating, nutrient sensing is crucial for fungal adaptation and survival and dedicated GPCRs for carbohydrate sensing have been described in many fungal species. A prime example is the glucose-sensing G-protein-coupled receptor 1 (Gpr1) from *S. cerevisiae* [9]. Homologs of these GPCRs have been identified in many ascomycetes most of which signal via the cAMP pathway. As a consequence, cAMP levels often define morphological adaptations such as the transition between yeast and hyphal growth forms (Fig 1b). Intriguingly, Gpr receptors do not exclusively recognize nutrients, but fulfill roles in prey recognition and subsequent cellular adaptations such as trap formation, as recently demonstrated for Gprs in the nematode-trapping fungi *Arthrobotrys oligospora* and *Arthrobotrys flagrans* [10,11] (Fig 1a).

In addition to fungal GPCRs required for pheromone and nutrient sensing, GPCRs of the Pth11-like class play important roles during the establishment of pathogenic interactions. The first member of this class was originally discovered in the rice blast fungus *Magnaporthe grisea* [12,13], and homologs have since been shown to mediate host interactions in *Fusarium graminearum* [14,15] as well as nematode prey recognition by *Arthrobotrys spp.* [10,11]. Recent studies have revealed that Pth11-like receptors are widely distributed across Pezizomycotina fungi, including many agriculturally important plant pathogens [3].

## GPCR-mediated signaling contributes to lichen biology

GPCRs do not only mediate interactions with multicellular hosts or nematode prey but likely play important roles in shaping and maintaining fungal microbial communities in diverse contexts [6]. Pth11-like GPCRs are not exclusive to pathogens but are also highly overrepresented in fungi with different lifestyles, including lichen-forming fungi [3]. Considering their conservation, it is tempting to speculate that GPCRs also contribute to cellular communication during the establishment and maintenance of lichens (Fig 1a). For example, the lichen-forming fungus *Cladonia grayi* has an expanded set of Pth11-type receptors along with five divergent Gα-subunit paralogs, which are also differentially induced during *in vitro* co-cultivation with the alga *Asterochloris glomerata* [16]. Additionally, downstream signaling components involved in GPCR-mediated pathways are also induced in *C. grayi* in presence of the photobiont partner.

Like some fungal pathogens, lichen-forming fungi may undergo morphological adaptations during the formation of a structured lichen thallus. Notably, *Umbilicaria muehlenbergii*, which is one of the few proposed lichen-forming fungi that can be cultured in isolation, displays a dimorphic growth phenotype [17]. The switch from its yeast form to (pseudo)hyphal growth can be triggered by nutrient starvation or contact with the compatible photobiont *Trebouxia jamesii* and is mediated by G-protein signaling via the cAMP-PKA and UMP1 MAPK pathways [17,18].

While the precise roles of GPCRs in lichen symbioses remain largely unexplored, their conservation, expansion, and dynamic regulation strongly suggest that they are key players in the establishment and maintenance of these complex microbial communities.

## Diversification of fungal GPCRs results in the recognition of alternative ligands and novel signaling mechanisms

Fungal GPCRs detect a wide range of signals including hormones, proteins, nutrients, ions, surfaces, and light but it is often unclear which specific ligand is recognized by a particular GPCR. It is, therefore, crucial to identify cognate GPCR/ligand pairs to fully understand their roles in development, metabolism, and symbiosis [1,5].

The best studied GPCR/ligand pairs are the pheromone mating receptors of the Ste2/Ste3-class that recognize peptide pheromones (termed α- and a in *S. cerevisiae*) which have been identified and characterized in many fungal species (Fig 1c). More recently, research on *Candida albicans* suggested that pheromone receptors can recognize signals beyond their cognate pheromones, triggering phenotypical changes like biofilm formation, with possible implications for host adaptation [19]. Although the chemical nature of these signals remains unknown, these observations indicate that pheromone receptors might have a broader ligand spectrum and play roles beyond mating.

Gpr class carbohydrate receptors, including Gpr1 of *S. cerevisiae* (ScGpr1), recognize glucose as well as structurally related sugars [20]. The *A. oligospora* Gpr1 homologs Gpr2 and Gpr3 as well as GprC in *A. flagrans* have diversified their ligand spectrum and recognize the nematode hormone ascaroside (Fig 1c and 1d), although at least GprC still binds glucose [10,11]. Strikingly, these receptors show a high structural similarity with the receptor SRBC-66 from their ‘prey’ *Caenorhabditis elegans* with shared residues in their ligand binding pockets, suggesting a common ancestral origin [11]. Furthermore, upon prey recognition GprC displays a novel signal transduction mechanism, localizing to both plasma and mitochondrial membranes thereby directly linking ligand binding to mitochondrial activity, which is required to initiate nematode preying [10]. Gpr-like receptors might have even further diversified, as GprC of *Aspergillus ochraceus* is able to detect 9-hydroxyoctadecadienoic acid, an oxylipin formed from the oxidation of linoleic acid [21], which structurally resembles the fatty acid-derived side chains found in ascarosides, suggesting a possible convergence in ligand recognition despite differing biosynthetic origins.

GPCR-mediated carbohydrate sensing likely also evolved in the context of other fungal symbioses. The chemical compounds released by photo- and mycobionts during lichen formation include well-characterized GPCR ligands [22]. For instance, upon co-cultivation with the mycobiont *Endocarpon pusillum*, the photobiont *Diplosphaera chodatii* releases a range of carbohydrates, including glucose [23]. However, canonical carbon-sensing GPCRs are rare in the lichen-forming Lecanoromycetes [3], indicating that GPCRs from different classes may be involved in carbohydrate sensing in these fungi.

Collectively, these findings underscore the evolutionary adaptability of fungal GPCRs in ligand recognition and signaling mechanisms and the urgent need to expand the currently rather limited sets of GPCR/ligand pairs.

## Structural biology reveals new mechanistic concepts of fungal GPCRs

Despite the detailed knowledge on ligands of some fungal GPCRs, experimental structural information on ligand- and G-protein binding remains elusive. A notable exception is the Ste2-pheromone receptor of *S. cerevisiae*, whose structure was solved recently by high-resolution cryo-electron microscopy in complex with its cognate α-pheromone peptide ligand (Fig 1c) [24,25]. While Ste2 shares sequence homology with mammalian Rhodopsin GPCRs, it lacks the conserved G-protein activation motifs [24]. Structural mapping of its conformational states (ligand-free, agonist-bound, and antagonist-bound) clarified its activation mechanism, where α-pheromone binding induces conformational shifts that are different from Rhodopsin receptors. This activation mechanism reveals that fungal GPCRs have evolved features that are distinct from their mammalian counterparts complicating the knowledge transfer and highlighting the need for in-depth structural studies of fungal GPCRs. Intriguingly, Ste2 can also form a homodimer, associated with the unique capability of simultaneously coupling two G-proteins (Fig 1d) [24]. Notably, *A. oligospora* Gpr2 and Gpr3 heterodimerize upon ligand binding [11] but whether and how this dimerization influences signaling has yet to be investigated. Dimerization is also frequently observed for human GPCRs, suggesting that oligomerization mechanisms for signal amplification and modulation of GPCRs are conserved from fungi to humans.

## Genomic analyses, structural modeling and synthetic biology enable GPCR identification and characterization

Using a convolutional neural network analysis, a recent study identified a vast number of potential GPCRs across 1,357 fungal species (Fig 1, bottom) revealing several novel GPCR classes [3]. This study further demonstrated that Alphafold multimer [26] modeling can be used to predict GPCR/G-protein coupling. As few fungal GPCR/G-protein pairs have been discovered so far, and functional characterization based on reverse genetics remains complicated due to the central role of G-proteins in fungal signaling, this is a promising approach to reveal potential GPCR/G-protein pairings (Fig 1, bottom). In addition to these approaches, structure prediction might hold even more potential: GPCRs are characterized by their hallmark 7TM fold, which adopts a highly specific arrangement. Structural modeling of GPCRs may therefore improve GPCR identification, especially for highly divergent or cryptic receptors undetectable by sequence-based homology searches.

Despite their large diversity, most GPCRs-mediated signaling involves highly conserved intracellular signaling cascades via heterotrimeric G-proteins. This evolutionary conservation facilitated the development of *S. cerevisiae*-based biosensor systems to study fungal GPCRs and their peptide ligands (Fig 1, bottom) [27,28]. In these engineered *S. cerevisiae* strains, heterologous GPCR expression is coupled to a streamlined MAPK cascade, triggering the expression of a fluorescent reporter upon ligand binding. (Fig 1, bottom). These systems were successfully used to ‘deorphanize’, i.e., identifying activating ligands of human GPCRs, and represent an excellent resource for understanding the ligand diversity of identified fungal GPCRs. They are particularly valuable for studying GPCRs from fungal species that are difficult to manipulate genetically or exist within complex microbial communities, such as lichens, offering a powerful tool to explore newly identified fungal GPCRs.

## Conclusion and future perspectives

The field of fungal GPCR biology is rapidly evolving, yet many fundamental questions remain unanswered. Most of our current knowledge on the roles of fungal GPCRs is based on few model species, many of which are mammalian pathogens. However, considering their widespread occurrence and conservation in many fungal species with diverse lifestyles, it is likely GPCRs play important roles in other ecological contexts affecting the assembly, structure, and stability of complex fungal communities. One of the most pressing challenges is the identification of ligands for the vast array of orphan GPCRs found in fungal genomes. High-throughput screening methods, such as synthetic yeast systems, will be crucial for uncovering novel ligand classes beyond traditional nutrients and pheromones. Paired with structural modeling, these approaches represent powerful tools to revisit previously identified GPCRs offering new perspectives on GPCR/G-protein coupling and identifying components of the signaling pathways. Finally, fungal GPCRs hold great potential for antifungal development and high-resolution cryo-EM structures of fungal GPCRs are required to enable structure-guided drug design for antifungal therapies and agricultural applications.
